# Developmental stage-specific effects of Pim-1 dysregulation on murine bone marrow B cell development

**DOI:** 10.1186/s12865-016-0152-1

**Published:** 2016-06-10

**Authors:** Zhihui Xu, Kimberly A. Gwin, Yulin Li, Kay L. Medina

**Affiliations:** The Key Laboratory Pathobiology, Ministry of Education, Norman Bethune College of Medicine, Jilin University, Changchun, 130000 People’s Republic of China; Department of Immunology, College of Medicine, Mayo Clinic, 200 First Street SW, Rochester, MN 55905 USA; Department of Pathology, Norman Bethune College of Medicine, Jilin University, Changchun, 130000 People’s Republic of China

**Keywords:** B cell precursors, *Eμ-Pim1* transgene, *pim1-/-*, Flt3, Hoxa9, B cell development, Lymphopoiesis, Hematopoiesis

## Abstract

**Background:**

The serine threonine kinase Pim-1 has documented roles in hematopoietic progenitor and B cell precursor proliferation and survival. Pim-1 is a molecular target of the transcription factor Hoxa9. Previous studies showed that *Pim-1* deficiency phenocopied the hematopoietic progenitor defect in *hoxa9-/-* mice and forced expression of Pim-1 normalized the in vitro proliferation defect inherent to *hoxa9-/-* hematopoietic progenitors. Pim-1 is induced by cytokine signaling, including the early lymphoid/B lineage regulators Flt3 and IL-7, and expression levels were shown to influence the size of the B cell compartment in bone marrow (BM).

**Results:**

In this study, we sought to determine if transgenic expression of Pim-1, driven by the immunoglobulin enhancer, *Eμ*, was sufficient to rescue the lymphoid/B cell precursor defect in *hoxa9* or *flt3-ligand* (*flt3l*) deficient mice. Unexpectedly, expression of *Eμ − Pim1* exacerbated lymphoid progenitor deficiencies in *flt3l-/-*, and to a lesser extent, *hoxa9-/-* mice. Furthermore, *Eμ − Pim1* expression alone reduced early myeloid and lymphoid, but not erythroid, progenitors. In contrast, *Pim-1* deficiency had no significant effect on early lymphoid/B cell development through the Pre-Pro-B cell stage, but caused a significant reduction in IgM^−^ B cell precursors. Importantly, loss of *Pim-1* did not phenocopy *hoxa9*- or *flt3l*-deficiency on the lymphoid/early B cell progenitor pools.

**Conclusions:**

These experimental findings demonstrate that Pim-1 overexpression has developmental-stage-specific effects on B lymphopoiesis and myelopoiesis. Importantly, these suggest that *Pim-1* deficiency does not contribute significantly to the early lymphoid/B cell developmental deficiency in *hoxa9-/-* or *flt3l*-/- mice.

## Background

The steady-state production of B lineage lymphocytes in bone marrow is contingent on the developmental-stage-specific expression and combinatorial activities of multiple regulatory proteins including transcription factors, signaling molecules, microRNAs, and epigenetic modifiers. A critical early event in B cell development is lymphoid lineage specification within the multipotential progenitor (MPP) pool which we and others have shown is dependent, in part, on the homeodomain transcription factor Hoxa9 and signaling via the receptor tyrosine kinase Flt3 [1–4]. Germline knockout of Hoxa9 or Flt3-ligand largely phenocopy each other with respect to reductions in numbers of MPPs, the lymphoid progenitor subsets ALP and BLP, and B cell precursors in BM. Importantly, Hoxa9 and Flt3 signaling function synergistically to regulate a critical early checkpoint in lymphoid development, as combined loss of Hoxa9 and Flt3 signaling ablates the generation of lymphoid primed multipotential progenitors [4].

Pim-1 is a member of the Pim family of proto-oncogenes that encode a distinct class of serine/threonine kinases. Pim-1 was originally identified as a target for proviral activation in Moloney murine leukemia virus induced T cell lymphomas [5]. PIM proteins are widely expressed in most tissues and co-expression of family members has been found in various tissues [6]. The transcription of Pim family members is induced by a variety of cytokines and mitogens that transduce their primary signal through the JAK/STAT pathway [7]. A role for Pim-1 in B cell development was previously demonstrated through analysis of *pim1-/-* and *Eμ-Pim1* transgenic mice [8, 9]. *Pim1-/-* Pre-B cells exhibited impaired in vitro proliferation in response to IL-7 and stem cell factor (SCF) that was rescued by expression of a functional Pim-1 transgene [10]. In contrast, overexpression of Pim-1increased numbers of IL-7 + SCF responsive B cell colonies. These combined data provided the first evidence that Pim-1 was an important regulator of B lymphopoiesis in mice, and linked Pim-1 to the IL-7R signaling pathway.

Cytokine signaling plays an essential role in early lymphoid/B cell development. Threshold levels of Flt3 signaling are required for the proliferation, survival, and maintenance of MPPs competent to generate B cell precursors [1, 11]. Flt3 signaling is mediated by the Ras and STAT5 pathways [12]. A dominant negative form of Ras was shown to phenocopy the B lineage developmental block in *flt3-/-* mice, impairing the proliferation of common lymphoid progenitors and Pre-Pro-B cells. The same study showed that Ras promoted STAT5-dependent Pro-B differentiation by enhancing expression of IL-7Rα [12]. Pim-1 is induced downstream of Jak2/STAT5 signaling and has also been implicated in playing a role in the proliferation and/or differentiation of myeloid progenitors [13–15]. Importantly, a role for Pim-1 in regulation of the early lymphoid/B cell progenitor pool, prior to expression of CD45R/B220, has not been reported.

Functional studies have confirmed a role for Pim-1 in regulating hematopoietic stem cell (HSC) proliferation and survival. HSCs from *pim1-/-* mice exhibited impaired repopulating capacity in competitive transplantation experiments [16]. In vitro assays revealed decreased cytokine mediated cell growth and differentiation of hematopoietic progenitors [7]. In contrast, overexpression of human Pim-1 driven by *vav* hematopoietic regulatory elements and SV40 showed enhanced hematopoietic progenitor function in vitro and in vivo [16]. The hematopoietic defects exhibited by *pim1-/-* mice are strikingly similar to those in *hoxa9-/-* mice as loss of *hoxa9* also impaired the proliferation and repopulating ability of HSCs [17]. Consistent with this observation, *pim1* is a direct target of Hoxa9 [18]. Somatic ablation of *pim1*, *hoxa9*, or *flt3* causes select reductions in hematopoietic progenitor subsets and B cell precursors. However, an obligate role for Pim-1 in regulation of lymphoid and/or early B cell development has not been investigated. In this study we evaluated the role of Pim-1 in murine lymphoid lineage specification and B cell development through comparative flow cytometric analysis of *hoxa9-/- x Eμ-Pim1*Tg, *flt3l-/- x Eμ-Pim1*Tg, *Eμ-Pim1* transgenic, *pim1-/-*, *hoxa9-/-,* and *flt3l-/-* mice. Our experimental findings revealed that Pim-1 dysregulation has developmental-stage-specific effects on B lymphopoiesis and early myeloid, but not erythroid progenitors. Furthermore, we show that *Pim1-*deficiency is not the basis of the lymphoid or early B cell developmental defects in *hoxa9-/- or flt3l-/-* mice.

## Methods

### Mice

Wildtype C57Bl/6 mice were generated from our breeding colony. *Pim1-/-* and *Eμ − Pim1* transgenic mice have been previously described [10]. *Pim1-/-* mice were provided by Andrew S. Kraft and *Eμ − Pim1* transgenic mice were provided by Jung-Hyun Park and permission for both obtained from A. Berns. All mice evaluated in this study were 8–12 weeks of age. C57Bl/6, *EμPim-1*Tg, *hoxa9-/- x Eμ-Pim1*Tg, and *flt3l-/- x Eμ-Pim1*Tg mice were bred in the Mayo Clinic animal facility, and all animal experiments detailed in this study were conducted under guidelines established and approved by the Mayo Clinic Institutional Animal Care and Use Committee. PCR, using DNA isolated from tail clips, was performed using previously published primers to confirm the genotype of compound mice [19, 20].

### Flow cytometric analysis of BM hematopoietic progenitor subsets and B cell precursors

Methods for flow cytometry and progenitor isolation have been described [4]. Flow cytometric analysis was performed on the FACS-Canto or LSRII cytometers (BD BioSciences, San Jose, CA) and data analysis performed with FlowJo software (Tree Star, Ashland, OR). All antibodies used in this study were purchased from eBioScience or Biolegend. HSC/MPPs were resolved using the following combinations of antibodies: Lineage positive APC cocktail (CD3ε, Ter119, B220, Mac-1, Gr-1), CD34 FITC, Flt3 PE, Sca-1 PerCP-Cy5.5, CD150 PE-Cy7, c-kit APC-eflour 780 and 1.5×10^6^ million events collected for the analysis. MEPs, CMPs, and GMPs were resolved using the following combinations of antibodies: Lineage positive APC cocktail (CD3e, Ter119, B220, Mac-1, Gr-1), CD34 FITC, Flt3 PE, Sca-1 PerCP-Cy5.5, c-kit APC-eflour 780 and 1.5 × 10^6^ million events collected for the analysis. ALP and BLP were resolved using the following combinations of antibodies: Lineage positive FITC cocktail (CD3ε, CD8α, CD11c, NK1.1, Ly6C, Ter119, B220, Mac-1, Gr-1), Flt3 PE, IL-7R PE-Cy7, Ly6D AF647, c-kit APC-eflour 780, PDCA1 PerCP-eflour 710 and 2×10^6^ million events collected for the analysis. Pre-Pro-B and BCP subsets were resolved using the following combinations of antibodies: B220 FITC, AA4.1 PE, PDCA1 PerCP-eflour 710, CD19 Pe-Cy7, Ly6D AF647, and IgM APC-Cy7 and 500,000 to 1 × 10^6^ million events collected for the analysis. Red blood cells and cellular debris were excluded from the analysis with mononuclear cell and doublet exclusion gates. Absolute cell numbers were determined by multiplying population frequencies (total subset event count divided by total event count) times mononuclear cell count. Cell counts reflect numbers of cells per 4 hindlimb leg bones.

### Realtime RT-PCR analysis of *pim1* transcript levels in bone marrow progenitor subsets

Hematopoietic progenitor subsets were purified by cell sorting for RNA isolation, cDNA synthesis, and qPCR analysis as we previously described [21]. HSC/MPP were purified as Lin^−^ (see Lin^+^ cocktail above) c-kit^hi^ Sca-1^+^ Flt3^-lo^, LMPP as Lin^−^ c-kit^hi^ Sca-1^+^ Flt3^hi^, CLP as Lin^−^ c-kit^lo^ IL-7R^+^ Sca-1^+^ Flt3^+^, Pre-Pro-B as B220^+^ CD43^+^ CD19^−^ IgM^−^ (which includes a mix of Pre-Pro-B, NK, and pDCs), Pro-B as B220^+^ CD43^+^ CD19^+^ IgM^−^, Pre-B as B220^+lo^ CD43^−^ CD19^+^ IgM^−^, and IgM^+^ as B220^+hi^ CD43^−^ CD19^+^ IgM^+^. Realtime PCR was performed using a *pim1* taqman probe (Mm00435712_m1) and gene expression normalized to 18S RNA. All cDNA samples were assayed in triplicate. Relative transcript abundance was determined using the 2^-ΔΔCT^ method.

### Statistics

Statistical significance was determined using the Student-*t* test. Data are reported as standard error of the mean (SEM) and *p*-values less than 0.05 were considered significant.

## Results

### Forced expression of Pim-1 does not rescue the lymphoid/B lineage deficiency in *hoxa9-/-* or *flt3l-/-* mice

**Fig. 1:**
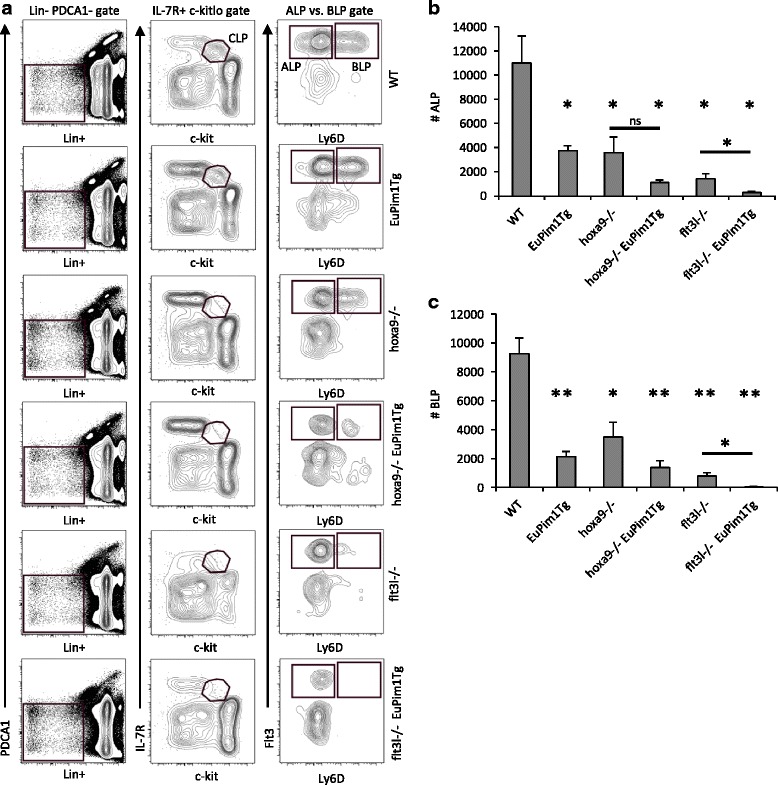
Forced expression of Pim-1 does not rescue the lymphoid deficiency in *hoxa9-/-* or *flt3l-/-* mice. **a** Flow cytometry profile and gating strategy for ALP and BLP in the various mouse strains. **b** Bar graph depicting absolute numbers of ALP in the various mice. **c** Bar graph depicting absolute numbers of BLP in the various mice. Data represents the mean ± SEM of 4–10 mice per group. A single asterisk * denotes *p* < 0.05 and ** denotes *p* < 0.005. To determine statistical significance, comparisons were made to WT, or between select comparators indicated by the line

**Fig. 2:**
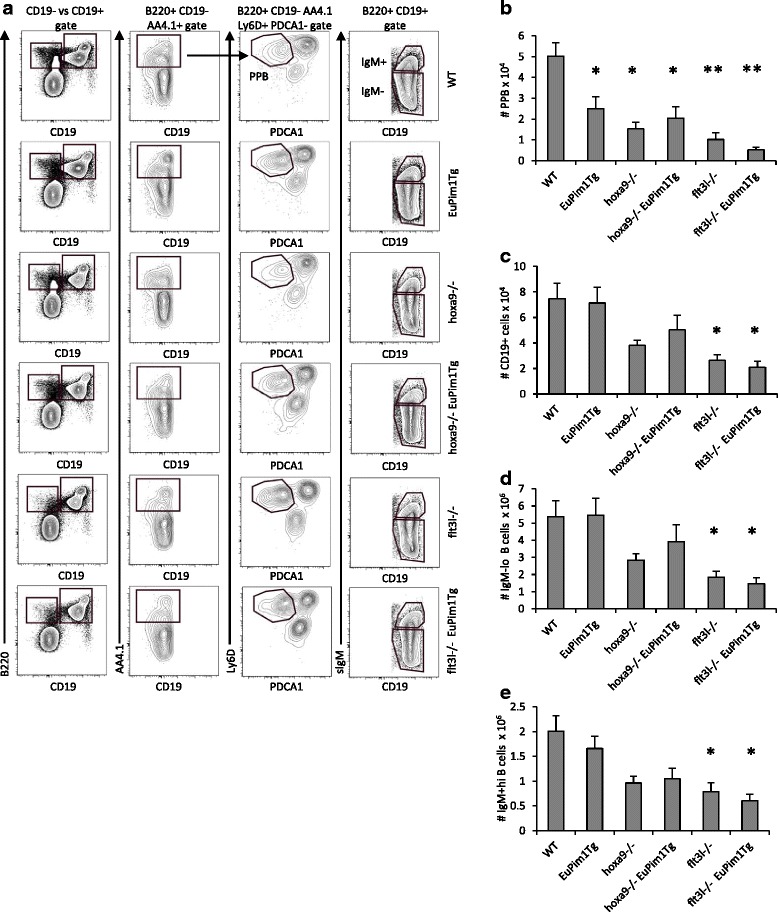
Effect of *Eμ-Pim1*Tg expression on B cell subsets in bone marrow. **a** Flow cytometric analysis of Pre-Pro-B, CD19^+^ cells, IgM^-lo^ B cells (which includes BCPs and recirculating B cells), and IgM^+hi^ naïve B cells in the various mouse strains. **b-e** Graphs depicting absolute numbers of (**b**) Pre-Pro-B cells, (**c**) CD19^+^ cells, (**d**) IgM^-lo^ B cells, and (**e**) IgM^+^ BCPs. Data represents the mean ± SEM of 4–10 mice per group. * denotes *p* < 0.05 and ** denotes *p* < 0.005

Gene-targeted ablation of *pim1*, *hoxa9*, or *flt3* causes reductions in select hematopoietic progenitor subsets and B cell precursors. Pim-1 is a molecular target of Hoxa9 and has been shown to be induced by Flt3 signaling [18, 22]. To determine if dysregulated expression of Pim-1 contributed to the lymphoid/B lineage precursor deficiency in *hoxa9-/-* or *flt3l-/-* mice, *hoxa9-/- x Eμ-Pim1*Tg and *flt3l-/- x Eμ-Pim1*Tg mice were generated. Transgenic expression of *Eμ-Pim1* had no significant effect on BM cellularity in *hoxa9-/-* or *flt3l-/-* mice (data not shown). *Hoxa9-/-* and *flt3l-/-* mice have significantly decreased numbers of Lin^−^ c-kit^lo^ IL-7R^+^ common lymphoid progenitors (CLPs) [4]. CLPs can be fractionated into Lin^−^ PDCA1^−^ IL-7R^+^ c-kit^lo^ Flt3^+^ Ly6D^−^ all lymphoid progenitors (ALP) and Lin^−^ PDCA1^−^ IL-7R^+^ c-kit^lo^ Flt3^+^ Ly6D^+^ B lymphoid progenitors (BLP) [23]. *Eμ* transgene driven expression of Pim-1 did not exacerbate the deficiencies in ALP or BLP in *hoxa9-/-* mice (Fig. 1a–c). However, *Eμ-Pim1*Tg expression did exacerbate the reductions in ALP and BLP in *flt3l-/-* mice (Fig. 1a–c). Expression of B220 distinguishes Pre-Pro-B cells from BLP [23]. Consistent with reductions in ALP and BLP, numbers of PDCA1^−^ Ly6D^+^ B220^+^ CD19^−^ Pre-Pro-B cells were reduced in *hoxa9-/- x Eμ-Pim1*Tg and *flt3l-/- x Eμ-Pim1*Tg mice compared to WT (Fig. 2a–b). However, in contrast to ALP and BLP, *Eμ-Pim1*Tg expression did not abrogate the Pre-Pro-B cell deficiency *hoxa9-/-* or *flt3l-/-* mice (Fig. 2a–b). The B lineage committed progeny of Pre-Pro-B cells are IgM^−^ B220^+^ CD19^+^ B cell precursors (BCPs). Unlike the negative impact of *Eμ-Pim1* transgene expression on CLP subsets and Pre-Pro-B cells, no statistically significant reduction in IgM^−^ B220^+^ CD19^+^ BCPs or total IgM^+^ B cells was found in *hoxa9-/- x Eμ-Pim1*Tg (Fig. 2a,c–d). However, reductions in IgM^−^ B220^+^ CD19^+^ BCP and total IgM^+^ B cell compartments were maintained in *flt3l-/-* and *flt3l-/- x Eμ-Pim1*Tg mice (Fig. 2a,c–d). These data reveal a previously uncharacterized inhibitory effect of *Eμ-Pim1*Tg expression on early lymphoid/B cell development.

### Impact of Pim1-deficiency versus *Eμ −* transgene driven expression of Pim-1 on BM lymphoid progenitors and BCPs

**Fig. 3:**
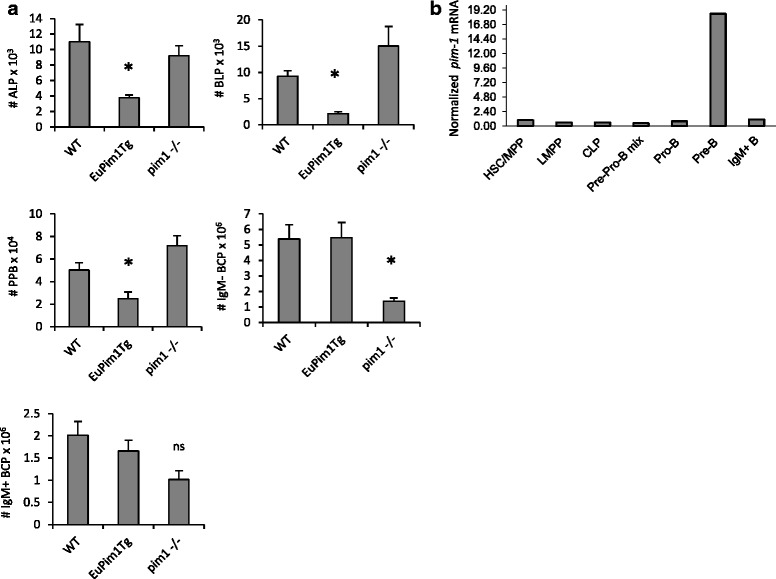
Impact of *Eμ-Pim1*Tg or *Pim1*-deficiency on lymphoid progenitor and B cell precursor subsets in bone marrow. **a** Bar graphs depicting absolute numbers of ALP, BLP, Pre-Pro-B, IgM^−^ B cell precursors, and IgM^+^ B cells. Data represents the mean ± SEM of 3–10 mice per group. * denotes *p* < 0.05 and ** denotes *p* < 0.005. **b** Realtime PCR analysis of *pim-1* transcript levels in sorted hematopoietic progenitor and B cell subsets with HSC/MPP designated as the comparator

A role for Pim-1 in B cell development was previously demonstrated from analysis of *pim1-/-* and *Eμ-Pim1* transgenic mice [10]. Our findings that *Eμ* transgene driven expression of Pim-1 abrogated lymphoid/early B cell development supported further evaluation of the effects of dysregulated expression of Pim-1 on development of this lineage. BM cells from WT, *pim1-/-*, and *Eμ-Pim1* transgenic mice were stained with combinations of antibodies to resolve ALP, BLP, Pre-Pro-B, IgM^−^ BCP, and IgM^+^ B cells. Comparison of BM mononuclear cell counts revealed no significant difference between WT and *Eμ-Pim1* transgenic mice, but a statistically significant reduction in *pim1-/-* mice (5.2×10^7^ ± 0.33, *n* = 3; *p* = 0.0055) compared to WT (7.9 ×10^7^ ± 1.3, *n* = 10). No statistically significant alterations in absolute numbers of ALP, BLP, or Pre-Pro-B were identified between WT and *pim1-/-* mice (Fig. 3a). The inverse was found in *Eμ-Pim1* transgenic mice wherein absolute numbers of phenotypic ALP, BLP, and Pre-Pro-B cells were significantly reduced compared to WT (Fig. 3a). Finally, while numbers of IgM^−^ BCP and IgM^+^ B cells in *Eμ-Pim1* transgenic mice were comparable to WT (Fig. 3a), we found that IgM^−^, but not IgM^+^, BCPs were reduced in *pim1-/-* mice [10]. CD19^+^ IgM^−^ BCPs constitute Pro-B and Pre-B cells. To gain further insight into the role of Pim-1 in hematopoietic development and B cell differentiation, we evaluated *pim-1* transcript levels in purified bone marrow progenitor subsets. As shown in Fig. 3b, abundant *pim-1* transcript expression was high exclusively in Pre-B cells across the B lineage developmental spectrum. Taken together, these data suggest that elevated levels of Pim-1 are inhibitory for lymphoid/B cell development prior to commitment to the B cell fate. On the other hand, loss of Pim-1 decreased IgM^−^ Pro-B/Pre-B cells, suggesting an essential role at this stage.

### Impact of Pim1-deficiency versus *Eμ −* transgene driven expression of Pim-1 on HSC, MPP, and erythroid/myeloid progenitors

**Fig. 4:**
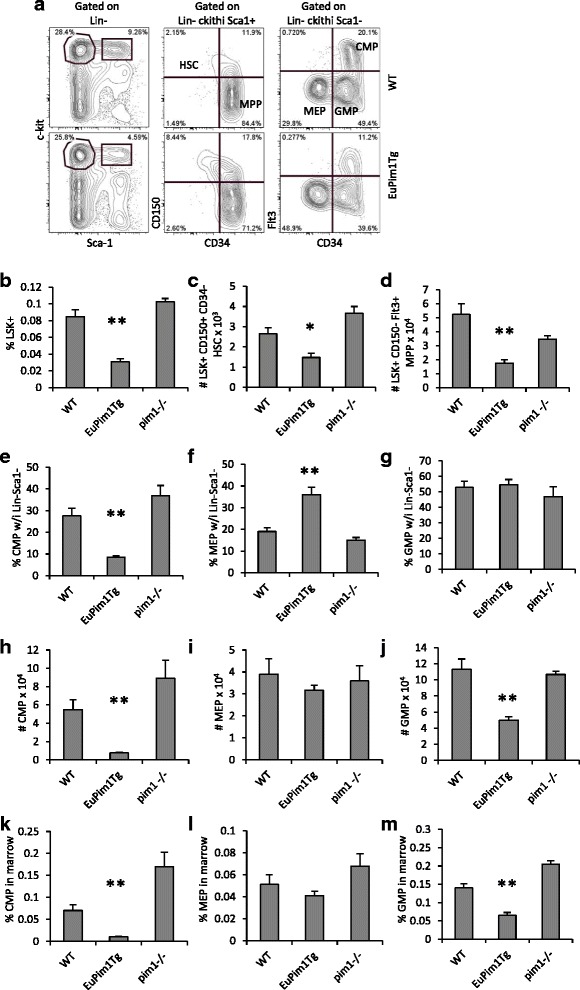
Impact of *Eμ-Pim1*Tg or *Pim1*-deficiency on primitive hematopoietic and myeloid-erythroid progenitor subsets in bone marrow. **a** Flow cytometry profile and gating strategy for HSC, Flt3+ MPP, CMP, GMP, and MEP. Bar graphs depict absolute numbers or frequencies of the various progenitor subsets. **b** Percentage of LSK+ in total BM. **c–d** Absolute number of HSC or MPP in BM. **e–g** Frequencies of CMP, MEP, or GMP within the Lin- ckithi Sca1- subset. **h–j** Absolute numbers of CMP, MEP, or GMP in BM. **k–m** Frequencies of CMP, MEP, or GMP in BM. Data represents the mean ± SEM of 3–10 mice per group. * denotes *p* < 0.05 and ** denotes *p* < 0.005

The *Eμ* enhancer is active very early in hematopoiesis, within LSK^+^ MPPs [11, 24]. A previous study reported increased numbers of LSK^+^ cells in *vav-Pim1Tg* mice, but a similar analysis has not been reported for *Eμ-Pim1*Tg mice [16]*.* To begin, we compared frequencies of LSK^+^ cells. In contrast to results reported for *vav-Pim1Tg* mice, frequencies and numbers of total LSK^+^ cells were significantly reduced in *Eμ-Pim1*Tg mice (Fig. 4a–b and data not shown). Next we compared frequencies and numbers of LSK^+^ CD150^+^ CD34^−^ HSC and LSK^+^ CD150^−^ Flt3^+^ MPP in *Eμ-Pim1*Tg mice. Absolute numbers of HSC and MPP were significantly reduced in *Eμ-Pim1*Tg mice (Fig. 4c–d). However, the reduction was attributed to the total decrease in LSK^+^ cells as percentages of CD150^+^ CD34^−^ HSC within the LSK^+^ compartment were increased in *Eμ-Pim1*Tg mice (6.23 ± 0.990 vs. 4.05 ± 1.07 %, *p* = 0.014, in *Eμ-Pim1*Tg vs. WT, respectively)*.* The same was true for the reduction in absolute numbers of MPP as percentages CD150^−^ Flt3^+^ within the LSK^+^ compartment was not significantly reduced (73.7 ± 4.53 vs. 80.1 ± 3.72 %, *p* = 0.064, in *Eμ-Pim1*Tg vs. WT, respectively)*.* The reduction in LSK^+^ cells in *Eμ-Pim1*Tg mice could influence numbers of erythroid/myeloid progenitors. Frequencies of Lin^−^ ckit^hi^ Sca1^−^ cells were not altered in *Eμ-Pim1*Tg mice (Fig. 4a). However, significant alterations in frequencies and absolute numbers of CMP and GMP, but not MEP, were documented *Eμ-Pim1*Tg mice (Fig. 4a,e–m). No significant alterations in frequencies or numbers of LSK^+^, HSC, MPP, CMP, MEP, or GMP were observed in *pim1-/-* mice, compared to WT (Fig. 4). Taken together, these data show that *Eμ-Pim1*Tg driven expression of Pim-1, but not *Pim1*-deficiency, reduces the myeloid but not the erythroid/megakaryocytic progenitor pools.

### Loss of Pim-1 does not phenocopy *hoxa9-* deficiency on the lymphoid/early B cell progenitor pools

**Fig. 5:**
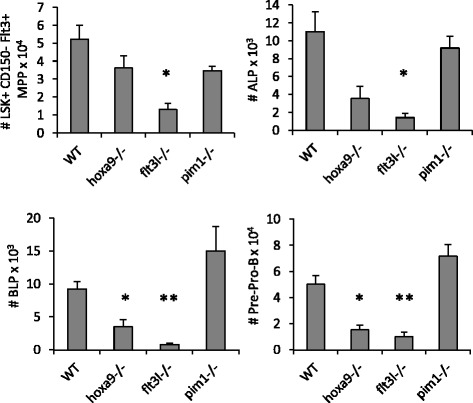
Comparison of *Hoxa9-*, *Fl3tl-*, and *Pim1-*deficiencies on hematopoietic progenitor subsets and Pre-Pro-B cells. Bar graphs depict absolute numbers of the various progenitor subsets. Data represents the mean ± SEM of 3–4 mice per single knockout and 10 WT mice. * denotes *p* < 0.05 and ** denotes *p* < 0.005

Pim-1 is a Hoxa9 target gene and forced expression of Pim-1 rescued the in vitro proliferation of *hoxa9-/-* BM progenitors [18]. We reasoned that if *Pim1*-deficiency is the basis of the hematopoietic progenitor defect in *hoxa9-/-* mice, then *pim1-/-* mice should phenocopy, at least in part, *hoxa9-/-* progenitor defects in BM. *Hoxa9-/-* and *pim1-/-* mice shared statistically significantly increased frequencies of HSCs. However, while *hoxa9-/-* and *flt3l-/-* mice shared similar reductions in CMP, GMP, ALP, BLP, and Pre-Pro-B cells, consistent with our previous observation, loss of *Pim-1* did not phenocopy *Hoxa9*-deficiency (Fig. 5) [4, 23].

## Discussion

Previous studies have implicated a role for Pim-1 in regulation of B cell development in BM and *pim-1* is a Hoxa9 target gene [10, 18]. Furthermore, *hoxa9-* and *pim1-*deficient mice share similar hematopoietic phenotypes and forced expression of Pim-1 rescued the in vitro proliferation defect in *hoxa9-/-* BM progenitors [17, 18]. To determine if forced expression of Pim-1 was sufficient to restore the lymphoid progenitor/B cell precursor defect in *hoxa9-/-* or *flt3l-/-* mice, we generated *hoxa9-/- Eμ-Pim1*Tg mice and *flt3l-/- Eμ-Pim1*Tg mice. Unexpectedly, *Eμ* driven expression of Pim-1 exacerbated the deficiencies in ALP and BLP in *flt3l-/-,* but not *hoxa9-/-* mice. The detrimental effect in *flt3l-/- Eμ-Pim1*Tg mice was specific to the ALP and BLP stages of B cell differentiation, as the *Eμ-Pim1*Tg did not further exacerbate the B cell deficiencies in *flt3l-/-* mice from the Pre-Pro-B through the IgM^+^ stages. The negative effect of *Eμ-Pim1*Tg expression was not restricted to the ALP and BLP compartments. We previously showed that *Eμ* is active from a very early stage in hematopoiesis and here we also show that *Eμ-Pim1*Tg expression caused significant reductions in numbers of HSCs, MPPs, CMPs, and GMPs suggesting that Pim-1 expression levels must be carefully controlled during early hematopoietic differentiation [11, 24]. In contrast, loss of Pim-1, which we hypothesized might have deleterious consequences on the early lymphoid/B cell progenitor pools, was dispensable for hematopoiesis with the exception of IgM^−^ BCPs [10]. Consistent, with our findings, realtime PCR of *pim-1* transcription across the hematopoietic spectrum, showed significant levels limited to BCPs at the Pre-B cell stage. Finally, through direct comparative hematopoietic progenitor subset analysis of *hoxa9-/-*, *flt3l-/-*, and *pim1-/-* mice, we suggest that loss of Pim-1 is unlikely to contribute significantly to the hematopoietic progenitor defects in either *hoxa9-/-* or *flt3l-/-* mice. Taken together, these comprehensive flow cytometric analyses demonstrate very select lineage and developmental-stage-specific consequences of dysregulated expression of Pim-1 expression on BM hematopoietic progenitor and B lineage subsets.

The serine threonine phosphokinase Pim-1 is highly expressed in hematopoietic cells in mouse and man [20, 25]. The *pim-1* gene was originally discovered as a common insertion site in MoMuLV-induced T cell lymphomas and later found in B-cell lymphomas and erythroleukemias. Overexpression of Pim-1 driven by the *Eμ* enhancer was shown to lead to a low incidence of T cell lymphomas and increased sensitivity to chemically induced T cell transformation. In the B lineage, overexpression of *Eμ −* Pim-1 facilitated establishment of B cell progenitor cell lines blocked at the Pre-B cell stage of differentiation [10]. The same study reported that marrows with the highest Pim-1 expression had a reduction in M-CSF responsive cells, and suggested the result could be due to impaired macrophage differentiation or on a macrophage progenitor. Our immunophenotypic analysis of *Eμ-Pim1* transgenic mice provides insight into the reductions in M-CSF responsive cells as we identified significant reductions in frequencies and absolute numbers of CMP and GMP.

PIM kinases are short-lived proteins induced at the level of transcription upon mitogenic stimulation [26]. They do not have regulatory domains and are constitutively active when expressed. Under physiologic conditions, their short half-life limits their activity [27]. Many cytokines that induce *pim* gene transcription act through the JAK/STAT pathway. The *pim1* promoter has binding sites for STAT3 and STAT5 [28, 29]. Pim-1 protein then, in turn, functions to inhibit the JAK/STAT pathway by binding and activating SOCS proteins [30]. SOCS proteins support cytokine signaling through the Ras/MAPK pathway by binding the Ras inhibitor RasGAP [30]. Interestingly, dysregulated Ras/SOCS function mediated by overexpression of Pim-1 provides a plausible cellular mechanism for the impaired lymphoid/early B lineage development exhibited by *Eμ − Pim1*Tg mice. Ras proteins are involved in regulating proliferation and differentiation of various cell types in a tissue specific manner. Pertinent to this study, mice expressing a dominant negative form of human H-Ras have a severe block in B cell development at the Pre-Pro-B to Pro-B transition [31]. Li, et al., previously showed that a Flt3/Ras dependent signal governs lymphoid progenitor (ALP + BLP) and Pre-Pro-B proliferation [12]. In addition, they showed that Flt3/Ras suppresses expression of *socs2* and *socs3*. They concluded that Flt3 activation of Ras primes B cell development by inducing a state of STAT5 responsiveness, a key event downstream of IL-7R signaling in lymphoid progenitors leading to induction of the B cell fate specification and commitment factor EBF1. Sustained expression of Pim-1 would be predicted to stabilize SOCS expression, impairing STAT5-mediated induction of *ebf1*, suppressing EBF1-directed B cell differentiation. Indeed, this hypothetical molecular explanation is consistent with our findings that the Flt3 dependent ALP and BLP stages are exquisitely sensitive to dysregulated Pim-1 in *flt3l-/-* mice. It also suggests that it is unlikely that elevated levels of Pim-1 are inhibitory for lymphoid/B cell development by accelerating B cell differentiation, given the critical role for STAT5/EBF1 in this process.

Pim kinases act as oncogenes by promoting cell cycle progression by phosphorylating and down-regulating the cyclin dependent kinase inhibitor p27Kip1. Pim kinases also have the ability to suppress *p27kip1* transcription through phosphorylation and inactivation of forkhead transcription factors, including FoxO1 and FoxO3 [32]. Pim-1 inactivation of FoxO1 would have deleterious effects on B cell fate determination as FoxO1 is an integral member of a global network of transcription factors, including E2A and EBF1 [33]. Importantly, *FoxO1*-deficient mice also have an arrest in B cell development at the CLP stage, specifically at the BLP stage, and phenocopy *EBF1*-deficiency [34]. Importantly, *FoxO1*-deficient BLPs have increased transcripts for *socs2*, reminiscent of Pim-1 overexpression [30]. Based on our immunophenotyping results, together with previous phenotypic, molecular, and cellular data in the literature database, we hypothesize that dyregulated expression on Pim-1, driven by *Eμ*, impairs lymphoid/early B cell differentiation through upregulation of SOCS proteins and inactivation of FoxO1. Upregulation of SOCS proteins inhibits STAT5 mediated B cell differentiation leading to upregulation of EBF1. Inactivation of FoxO1 impairs the global transcriptional network orchestrated by E2A, EBF1, and FoxO1 that directs the early program of B lineage gene expression, requisite for B cell differentiation. In contrast, loss of Pim-1, would be predicted to suppress SOCS protein expression and stabilize FoxO1, favoring B cell developmental potential, providing an explanation for the slight, but not statistically significant, increase in BLP and Pre-Pro-B cells we observed in *pim1-/-* mice. We note that additional studies are required to determine if this molecular explanation is valid.

The goal of this project was to investigate the role of Pim-1 in early lymphoid/B cell development. However, we also found reductions in numbers of HSC, MPP, CMP, and GMP in *Eμ − Pim1*Tg mice. An, et al., reported an increase in frequencies and numbers of LSK^+^ cells in *vav-Pim1Tg* mice, whereas we report decreased frequencies and numbers of LSK^+^ cells in *Eμ − Pim1*Tg mice [16]. The overall decrease in LSK^+^ cells was the basis of the decreased numbers of HSCs and MPPs, as frequencies of CD150^+^ CD34^−^ immunophenotypic HSC within the LSK^+^ compartment were actually increased in *Eμ − Pim1*Tg mice. We believe it unlikely that *Eμ − Pim1*Tg expression has any functional consequence on HSC biology for two reasons. First, we previously showed that *Eμ* driven reporter expression is confined to CD27^+^ MPPs within the LSK^+^ compartment and HSCs are CD27^−^ [11, 24, 35]. Second, the frequencies of CD150^+^ CD34^−^ HSCs were substantially increased in *Eμ − Pim1*Tg mice.

The cellular and/or molecular basis of the inhibitory consequence of *Eμ − Pim1*Tg expression on numbers of Flt3^+^ MPPs in noteworthy, as Flt3^+^ MPPs are the precursors of CMP and CLP and these hematopoietic subsets were the most sensitive to *Eμ − Pim1*Tg expression. *Eμ − Pim1*Tg expression did not impact Flt3^+^ CMP or CLP by altering the expression levels of Flt3. However, Flt3^+^ CMP and CLP were particularly sensitive to *Eμ − Pim1*Tg expression. These findings suggest that overexpression of Pim-1 might negatively impact signaling networks downstream of Flt3 stimulation in Flt3^+^ progenitor subsets, impairing either their proliferation or survival. Regardless of the mechanism, differentiating CMP and CLP overcome the inhibitory effects and their downstream progeny are refractory to sustained expression of Pim-1. Overexpression of Pim-1 in myeloid progenitors is likely limited by inactivation of *Eμ* due to lack of expression of critical activators necessary to maintain enhancer activity. It is interesting to note that Pim-1 driven by *vav* regulatory elements was low in mature myeloid cells [16].

Finally, although our flow cytometry analysis revealed that *Pim1-*deficiency does not provide a molecular explanation for the lymphoid/B cell deficiency in *hoxa9-/-* mice, *pim1-/-* and *hoxa9-/-* mice do share similar phenotypic and functional HSC defects. These observations and reported findings underscore developmental stage specific requirements for Hoxa9 and Pim-1 in hematopoietic development.

## Conclusions

The goal of this study was to determine the requirement for Pim-1 in early lymphoid/B cell development. We show that controlled expression of Pim-1 is important in lymphoid/B cell development. High levels of Pim-1 are inhibitory while Pim-1 is dispensable for establishment of lymphoid progenitor and Pre-Pro-B cell pools.

## Abbreviations

ALP, all lymphoid progenitors; BCP, B cell precursors; BLP, B lineage progenitors; BM, bone marrow; CMP, common myeloid progenitor; flt3l, Flt3-ligand; GMP, granulocyte-macrophage progenitor; HSCs, hematopoietic stem cells; MEP, megakaryocyte-erythroid progenitor; MPP, multipotent progenitor; PPB, Pre-Pro-B; Tg, transgenic.
